# Functional reorganization of the contralesional precentral gyrus following acute subcortical ischemic stroke and its association with gene expression profile

**DOI:** 10.1186/s12984-026-02048-w

**Published:** 2026-06-12

**Authors:** Xueqin Jiang, Qiuhong Lu, Yang Liu, Huachun Huang, Yuansheng Bi, Zhijian Liang

**Affiliations:** Department of Neurology, The First Affiliated Hospital of Guangxi Medical University, Nanning, China

**Keywords:** Subcortical ischemic stroke, Motor recovery, Resting-state fMRI, ALFF, Functional connectivity, Gene expression

## Abstract

**Background:**

Motor recovery after ischemic stroke involves complex functional reorganization, yet the underlying molecular and cellular mechanisms remain poorly understood. This study integrated longitudinal neuroimaging and brain-wide transcriptomic data to characterize the functional dynamics and their gene-expression correlates during motor recovery following subcortical ischemic stroke.

**Methods:**

We recruited 34 patients with acute right subcortical ischemic stroke and 32 age- and sex-matched healthy controls. All participants underwent baseline resting-state functional magnetic resonance imaging, with 22 patients completing a 3-month follow-up scan. Spontaneous neural activity was assessed using the amplitude of low-frequency fluctuations (ALFF), followed by seed-based whole-brain functional connectivity (FC) analysis from regions with longitudinal ALFF differences. We then applied partial least squares (PLS) regression to spatially correlate longitudinal ALFF changes with transcriptomic data from the Allen Human Brain Atlas, identifying a gene expression profile spatially associated with these ALFF changes. These genes were subsequently subjected to functional enrichment and cell-type specificity analyses.

**Results:**

Compared with healthy controls, acute-stage stroke patients showed significantly decreased ALFF in the contralesional precentral gyrus. At 3-month follow-up, ALFF in this region significantly increased, accompanied by strengthened interhemispheric FC with its ipsilesional homologue. Critically, these longitudinal changes in ALFF and interhemispheric FC were significantly correlated with motor recovery. Based on PLS regression, we further identified a specific gene expression profile spatially correlated with the observed ALFF changes. This gene set was specifically enriched in excitatory and inhibitory neurons and was primarily involved in synaptic structure and signaling.

**Conclusions:**

By linking macroscale imaging dynamics with microscale molecular features, this study demonstrates that the contralesional precentral gyrus plays a supportive role in motor recovery during the subacute phase of subcortical ischemic stroke, with neuronal synaptic plasticity as a potential mechanism. Collectively, these findings inform stage-specific strategies to target interhemispheric inhibition.

**Supplementary Information:**

The online version contains supplementary material available at 10.1186/s12984-026-02048-w.

## Introduction

Stroke, the second leading cause of death worldwide and a major contributor to adult long-term disability, poses a growing socioeconomic and public health burden [1–3]. Ischemic events account for the majority of strokes and frequently lead to persistent motor deficits that severely affect patients’ quality of life [4, 5]. Following a stroke, the brain undergoes a dynamic process of remodeling, involving not only peri-infarct regions but also remote areas, including the contralesional hemisphere [6]. The role of the contralesional hemisphere in post-stroke motor recovery, however, remains debated [7]. While the traditional “interhemispheric competition” model posits that contralesional overactivity inhibits the injured hemisphere and impedes recovery [8], accumulating evidence suggests that activation of the contralesional motor cortex, particularly the precentral gyrus, may represent a critical functional adaptation, especially in patients with limited structural reserve. This adaptation may contribute to motor control of the paretic limb via alternative descending pathways originating from the contralesional hemisphere, thereby serving a compensatory function [9–14]. Nevertheless, the dynamic trajectory of involvement of the contralesional precentral gyrus throughout recovery, and its underlying biological basis, require further elucidation through carefully designed longitudinal studies.

Resting-state functional magnetic resonance imaging (rs-fMRI), a non-invasive tool for in vivo investigation of brain function, provides a vital means for probing spontaneous neural activity and the brain’s intrinsic functional architecture [15]. Among various analytical metrics, the amplitude of low-frequency fluctuations (ALFF), which quantifies the intensity of local spontaneous neural activity, has been widely applied to study neurological and psychiatric disorders [16–19]. In stroke patients, ALFF alterations in both ipsilesional and contralesional hemispheres have been associated with motor recovery, suggesting its potential as a biomarker for predicting motor outcomes [20–23]. Complementarily, functional connectivity (FC) measures the temporal synchronization of neural activity between spatially separated brain regions, effectively revealing functional network reorganization post-stroke, such as changes in interhemispheric connectivity [24, 25]. Integrating ALFF with FC offers a system-level perspective for examining post-stroke brain plasticity.

Current stroke research primarily focuses on macroscopic neuroimaging phenotypes, often overlooking the molecular mechanisms underlying post-stroke plasticity. The emerging field of neuroimaging-transcriptomics addresses this gap by spatially correlating gene expression data with neuroimaging patterns derived from in vivo human brains. The Allen Human Brain Atlas (AHBA) is currently the most comprehensive whole-brain transcriptomic dataset mapped to stereotaxic space, providing high-resolution coverage of over 20,000 genes across the human brain [26, 27]. Using this atlas, researchers have successfully uncovered molecular correlates in brain disorders such as Parkinson’s disease, epilepsy, and depression [28–31], demonstrating its potential to inform molecular explanations for complex brain phenotypes. However, its application to stroke recovery research has remained largely unexplored, representing a promising direction for gaining novel molecular-level insights.

Therefore, this prospective longitudinal study investigated subcortical post-stroke motor recovery by integrating macroscale neuroimaging with brain-wide transcriptomic data. We hypothesized that neural activity in the contralesional precentral gyrus would demonstrate a pattern of initial suppression followed by enhancement, with the latter accompanied by strengthened interhemispheric connectivity, and that this macroscale plasticity pattern would correlate with a specific gene expression profile. To test this, we first analyzed the changes in spontaneous neural activity (indexed by ALFF) and whole-brain FC from baseline to 3-month follow-up in patients with subcortical ischemic stroke (SIS). We then correlated the longitudinal ALFF change patterns with AHBA-derived gene expression data to uncover the gene sets associated with these changes, followed by functional enrichment and cell-type specificity analyses. This work aims to provide novel neurobiological insights into subcortical post-stroke motor recovery, with potential implications for future rehabilitation strategies.

## Materials and methods

### Participants

This prospective longitudinal observational study was approved by the Ethics Committee of the First Affiliated Hospital of Guangxi Medical University (No. 2021[KY-E-184]) and conducted in accordance with the Declaration of Helsinki. All participants provided written informed consent after being fully informed of the study details. We consecutively enrolled patients with first-ever acute right-hemispheric SIS from the Neurology Department. Inclusion criteria were: (1) SIS confirmed by diffusion-weighted imaging; (2) within 7 days of onset; (3) aged 30–80 years; (4) premorbidly right-handed; (5) presence of limb motor deficits (a score of ≥ 1 on the motor items of the National Institutes of Health Stroke Scale [NIHSS]). Exclusion criteria included: (1) history of neurological or psychiatric disorders; (2) severe aphasia, cognitive impairment, or auditory/visual deficits precluding assessment; (3) MRI contraindications; and (4) excessive head motion (translation > 2.5 mm or rotation > 2.5° in any direction) or poor image quality.

Simultaneously, we recruited age-, sex-, and education-matched healthy controls (HCs) who were right-handed and free of neurological or psychiatric disorders. Initially, 36 SIS patients and 37 HCs completed baseline assessment and MRI scanning. After excluding 7 participants (2 SIS, 5 HCs) due to excessive head motion or poor image quality, the final sample consisted of 34 SIS patients (22 completed the 3-month follow-up) and 32 HCs. Thus, in this study, the contralesional hemisphere consistently refers to the left hemisphere.

All SIS patients received standard post-stroke management throughout the study period, in accordance with the 2021 AHA/ASA guideline for secondary stroke prevention [32] and the AHA/ASA adult stroke rehabilitation guidelines [33]. This comprised universal secondary prevention medication (including antiplatelet agents and statins) and physical therapy as clinically indicated, ranging from bedside instruction to inpatient physiotherapy. No patients received any experimental, targeted, or device-based neuromodulation interventions.

### Clinical assessment

Neurofunctional assessments were conducted at baseline and at the 3-month follow-up using standardized scales. The NIHSS total score (range 0–42) was used to evaluate overall neurological deficits, with higher scores indicating greater impairment [34]. The Fugl-Meyer Assessment (FMA) served as the primary measure for motor function, assessing the upper (66 points) and lower (34 points) limbs, with higher total scores reflecting better motor recovery [35].

### MRI data acquisition

MRI data were acquired on a Siemens Prisma 3.0T scanner with a 64-channel phased-array head coil. During scanning, participants were instructed to remain awake, keep their eyes closed, and not think of anything in particular. High-resolution T1-weighted structural images were obtained using a magnetization-prepared rapid gradient-echo sequence (repetition time [TR] = 2300 ms, echo time [TE] = 2.98 ms, slice thickness = 1.0 mm, 176 slices) for spatial normalization and anatomical localization. We acquired rs-fMRI data using a gradient-echo echo-planar imaging sequence (TR = 2170 ms, TE = 30 ms, flip angle = 90°, slice thickness = 3.0 mm, 70 interleaved slices), with a total acquisition time of 6 min and 43 s.

### MRI data preprocessing

Preprocessing of rs-fMRI data was conducted using SPM12 (http://www.fil.ion.ucl.ac.uk/spm) and RESTplus V1.27 toolkit [36] (http://restfmri.net/forum/restplus) on the MATLAB R2017b platform (https://www.mathworks.com/products/matlab.html). The pipeline encompassed the following steps: (1) removal of the first 10 time points; (2) slice timing correction for inter-slice acquisition time differences; (3) head motion correction; (4) co-registration of T1-weighted structural images to the functional images, followed by tissue segmentation and then spatial normalization of the functional images to the Montreal Neurological Institute (MNI) space; (5) spatial smoothing with a 6 mm full-width at half-maximum Gaussian kernel; (6) linear detrending; (7) regression of nuisance covariates, including signals from white matter and cerebrospinal fluid, as well as the Friston 24 head motion parameters [37]; and (8) band-pass filtering (0.01–0.08 Hz) to isolate low-frequency fluctuations.

### ALFF calculation

The preprocessed time series underwent a Fast Fourier transform to obtain the power spectrum. The amplitude spectrum was then calculated as the square root of the power spectrum at each frequency. ALFF was subsequently defined as the average of this amplitude spectrum within the frequency range of 0.01–0.08 Hz [16]. To minimize inter-subject global variations, individual whole-brain ALFF maps were z-standardized (i.e., by subtracting the global mean and dividing by the standard deviation), resulting in zALFF maps for subsequent statistical analysis.

### FC calculation

Based on longitudinal ALFF changes from baseline to the 3-month follow-up, spherical seed regions with a 5-mm radius [38] were centered at the peak coordinates in the left precentral gyrus (MNI: -27, -24, 60) and the left paracentral lobule (MNI: -9, -33, 66). The mean time series of each seed region were extracted, and Pearson correlation coefficients were calculated between each seed and all other brain voxels. Correlation coefficients were then converted to z-values using Fisher’s z-transformation to generate individual-level zFC maps for subsequent analyses.

### AHBA gene expression data preprocessing

Gene expression data, comprising microarray expression profiles from 3,702 spatially unique samples across six adult donors (5 males, 1 female; mean age 42.5 years), were obtained from the AHBA [26] (http://human.brain-map.org). Using 58,692 probes, transcriptomic expression was assayed for 20,737 genes across regions, including the cerebral cortex, subcortex, brainstem, and cerebellum. As only two donors provided right-hemisphere samples, all subsequent analyses were restricted to the left hemisphere. Data were processed using the abagen toolbox [27] (https://github.com/rmarkello/abagen). The pipeline consisted of the following steps: (i) reannotating probes to standard gene symbols; (ii) intensity-based filtering to exclude probes expressed below background noise in more than 50% of samples across donors; (iii) selecting the probe with the most consistent pattern of regional variation across donors (i.e., differential stability) for each gene; (iv) mapping samples to the Human Brainnetome Atlas [39] using a 2-mm Euclidean distance threshold; (v) normalizing gene expression measures using a scaled robust sigmoid function across both genes and tissue samples to account for inter-individual differences and outlying values; (vi) averaging samples within the same region, separately for each donor and then across donors. This yielded a final expression matrix of 15,633 genes across 123 regions in the left hemisphere.

### Gene expression–neuroimaging association analysis

**Fig. 1:**
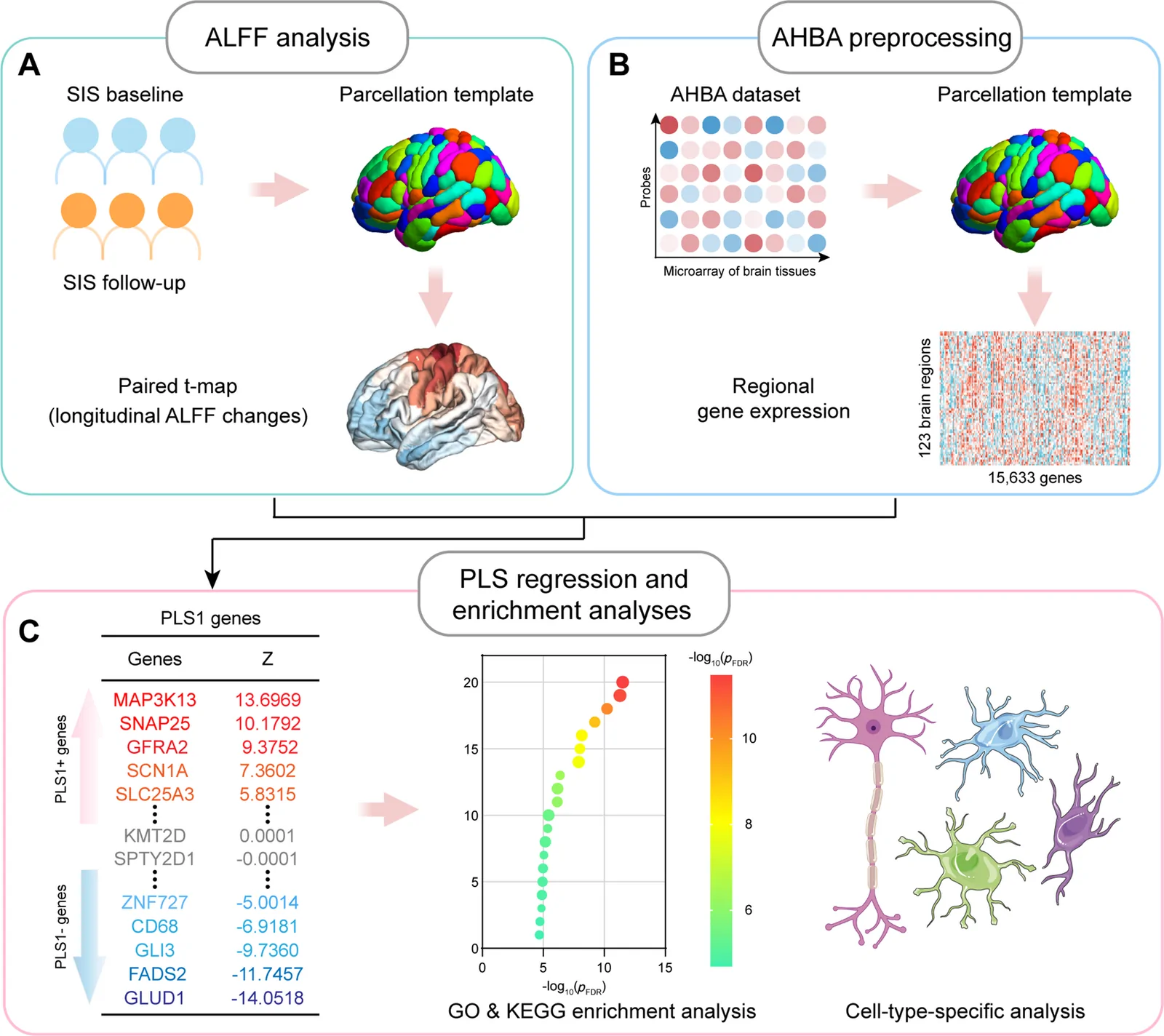
-

The specific workflow of the gene expression–neuroimaging association analysis is illustrated in Fig. 1. To explore the relationship between regional longitudinal ALFF changes and gene expression patterns in patients with SIS, we performed partial least squares (PLS) regression [40]. The longitudinal paired t-statistic map of ALFF across 123 left-hemisphere brain regions served as the dependent variable, and the expression levels of all 15,633 genes served as independent variables. The first PLS component (PLS1) was identified as the linear combination of gene expression that showed the strongest correlation with longitudinal ALFF changes. We conducted a permutation test (10,000 iterations) based on spherical rotations (spin test) to account for spatial autocorrelation, thereby constructing a valid null distribution for assessing whether the covariance captured by PLS1 exceeded chance levels [31]. Using 10,000 bootstrap resamples, we estimated the standard error of each gene’s PLS1 weight. Z-scores were calculated as weight divided by its bootstrapped standard error. Genes were ranked based on their Z-scores, and those with significant Z-scores (*p* < 0.05 after false discovery rate [FDR] correction) were further selected by |Z| > 5, resulting in two gene sets: positive-weighted genes (PLS1+, Z > 5) and negative-weighted genes (PLS1–, Z < −5) [41]. PLS1+ and PLS1– genes were positively and negatively correlated with longitudinal ALFF changes, respectively. We then assessed the spatial correlation between PLS1 scores and the paired t-statistic map of ALFF via Spearman’s correlation, with significance evaluated using a spin test to correct for potential confounding effects of spatial autocorrelation [42].

### Functional enrichment analysis

To characterize the biological functions, the PLS1+ and PLS1– gene sets were separately subjected to Gene Ontology (GO) enrichment analysis (including Biological Process, Cellular Component, and Molecular Function) and Kyoto Encyclopedia of Genes and Genomes (KEGG) pathway enrichment analysis via the Metascape online platform (https://metascape.org). Significant enrichment was defined by an FDR-corrected *p* < 0.05.

### Cell type assignment and transcriptional signature assessment of PLS1+ genes

To identify the distribution pattern of PLS1+ genes across specific brain cell types, we utilized marker gene sets for seven canonical cell types—excitatory neurons, inhibitory neurons, astrocytes, microglia, oligodendrocytes, oligodendrocyte precursor cells, and endothelial cells. These gene sets were obtained from Seidlitz et al. [43], who integrated data from five human postmortem cortical single-cell studies. For the PLS1+ gene set, we used permutation testing to determine the significance of cell type-specific gene overlap, with FDR correction applied (*p* < 0.05). Furthermore, to elucidate the biological functions of the overlapping genes in each cell type, GO and KEGG pathway enrichment analyses were conducted using the Metascape online platform (https://metascape.org), with FDR-corrected *p* < 0.05 considered significant.

### Statistical analysis

Statistical analyses were performed using GraphPad Prism (v9.0). For comparisons between baseline SIS patients and HCs, continuous data assessed for normality with the Shapiro-Wilk test were presented as mean ± standard deviation (compared using an independent-samples t-test) or median (interquartile range, IQR) (compared using a Mann-Whitney U test). Categorical variables were compared using the chi-square test. Longitudinal changes in clinical scores (baseline vs. 3-month follow-up) were analyzed using the Wilcoxon signed-rank test. A two-tailed *p* < 0.05 defined statistical significance for all tests.

For neuroimaging data, a two-sample t-test, controlling for age, sex, years of education, and mean framewise displacement (FD), was used to compare ALFF differences between baseline patients and HCs. Longitudinal ALFF/FC changes in patients between the 3-month follow-up and baseline were assessed using a paired t-test. All statistical maps, corrected for multiple comparisons using Gaussian random field (GRF) theory (voxel-level *p* < 0.005, cluster-level *p* < 0.05), were visualized using BrainNet Viewer [44] and MRIcron toolbox. Furthermore, we extracted longitudinal change values (Δ = follow-up − baseline) of ALFF/FC from significant brain regions. The correlations of these changes with changes in clinical scores were assessed using Pearson correlation (for ΔFMA) and Spearman rank correlation (for ΔNIHSS), with a Bonferroni-corrected significance threshold of *p* < 0.0042 (0.05/12).

## Results

### Demographic and clinical characteristics

The final analysis included data from 34 SIS patients (22 of whom completed the 3-month follow-up) and 32 HCs. No significant differences were observed between groups in age, sex, education, or mean FD (all *p* > 0.05) (Table 1). All 34 patients had right-hemispheric subcortical infarcts and presented with left-sided motor deficits. Among the 22 patients who completed follow-up, 20 (90.91%) presented with hemiparesis (weakness involving both upper and lower limbs), 1 had hemiplegia, and 1 had faciobrachial paresis. Lesions involved the basal ganglia in 15 patients (68.18%), the internal capsule in 14 (63.64%), and the corona radiata in 11 (50.00%), with many patients having involvement of multiple regions (see Supplementary Table 1 for individual details). As shown in Table 2, the median baseline NIHSS score was 4 (IQR 3–6), and the median baseline FMA score was 81.00 (IQR 72.00–85.75), which corresponds to a marked motor impairment. After 3 months, both measures improved significantly, with NIHSS reduced to 1.5 (IQR 0–4) and FMA increased to 96.50 (IQR 80.75–99.00) (both *p* < 0.0001).

**Table 1:** Participant characteristics

	SIS (*n* = 34)	HCs (*n* = 32)	*p*-value
Age (years)	56.35 ± 9.59 ^a^	54.31 ± 8.60 ^a^	0.3671 ^c^
Sex			
Male	25 (73.53)	18 (56.25)	0.1409 ^d^
Female	9 (26.47)	14 (43.75)	
Education (year)	12 (9–12.75) ^b^	12 (9–15) ^b^	0.5160 ^e^
Mean FD (mm)	0.13 (0.11–0.16) ^b^	0.13 (0.09–0.18) ^b^	0.9949 ^e^

a, mean ± standard deviation; b, median (interquartile range); c, independent samples t-test; d, chi-square test; e, Mann-Whitney U test; FD, framewise displacement; SIS, subcortical ischemic stroke; HCs, healthy controls

**Table 2:** Neurological scale scores

	Baseline (*n* = 22)	Follow-up (*n* = 22)	ΔFMA/|ΔNIHSS|	*p*-value
FMA score	81.00 (72.00–85.75)	96.50 (80.75–99.00)	12.50 (8.00–16.50)	< 0.0001
NIHSS score	4 (3–6)	1.5 (0–4)	2.5 (1.75–3)	< 0.0001

Data are presented as median (interquartile range). Within-group comparisons were performed using the Wilcoxon signed-rank test. FMA: Fugl-Meyer Assessment (total score, range 0–100); NIHSS: National Institutes of Health Stroke Scale (total score, range 0–42)

### Altered ALFF in patients with acute SIS

**Fig. 2:**
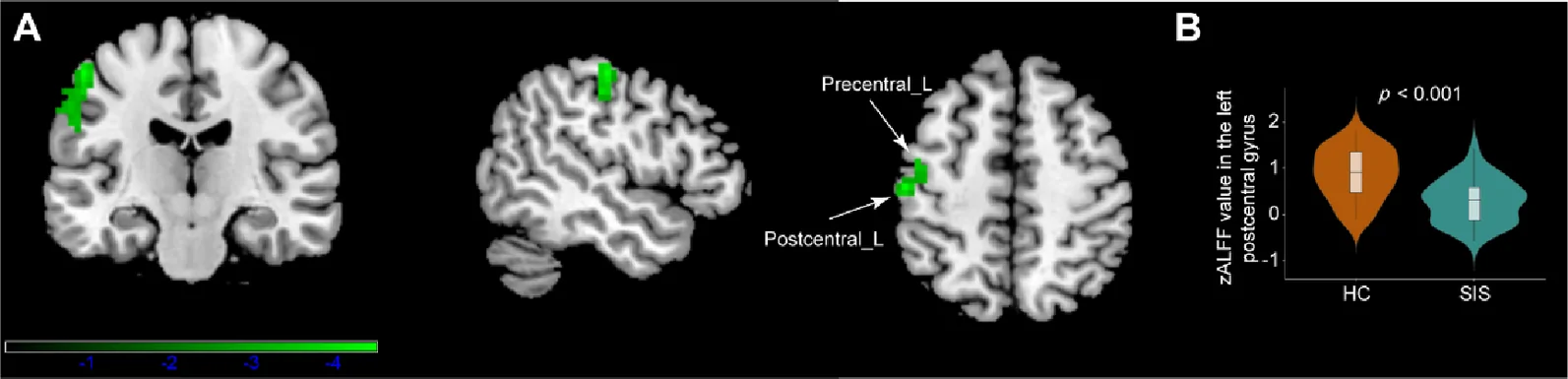
Baseline group differences in ALFF. The map highlights cortical regions central to our primary hypothesis (see Table 3 for cerebellar findings). **A** Brain regions showing significantly decreased ALFF in SIS patients compared with HCs at baseline. Statistical analysis was performed using a two-sample t-test with GRF correction (voxel-level *p* < 0.005, cluster-level *p* < 0.05). **B** Group comparison of mean ALFF values in the postcentral gyrus (extending to the precentral gyrus) between SIS patients and HCs (two-sample t-test)

Compared to HCs, acute SIS patients showed significantly decreased ALFF in the left postcentral gyrus (peak MNI coordinate: -51, -18, 54), extending to the left precentral gyrus, and increased ALFF in the bilateral cerebellar lobule VIII (GRF-corrected, voxel-level *p* < 0.005, cluster-level *p* < 0.05; Table 3; Fig. 2A, B).

**Table 3:** Brain regions with significant ALFF alterations between groups

Brain regions (AAL)	Number of voxels	MNI coordinate (x, y, z)	Peak T value
Postcentral_L	97	(-51, -18, 54)	-4.544
Cerebelum_8_R	168	(27, -39, -33)	4.318
Cerebelum_8_L	42	(-30, -39, -48)	4.3028

AAL, automated anatomical labeling; MNI, Montreal Neurological Institute; Postcentral_L, left postcentral gyrus; Cerebelum_8_R, right cerebellar lobule VIII; Cerebelum_8_L, left cerebellar lobule VIII

### Longitudinal changes in ALFF

**Fig. 3:**
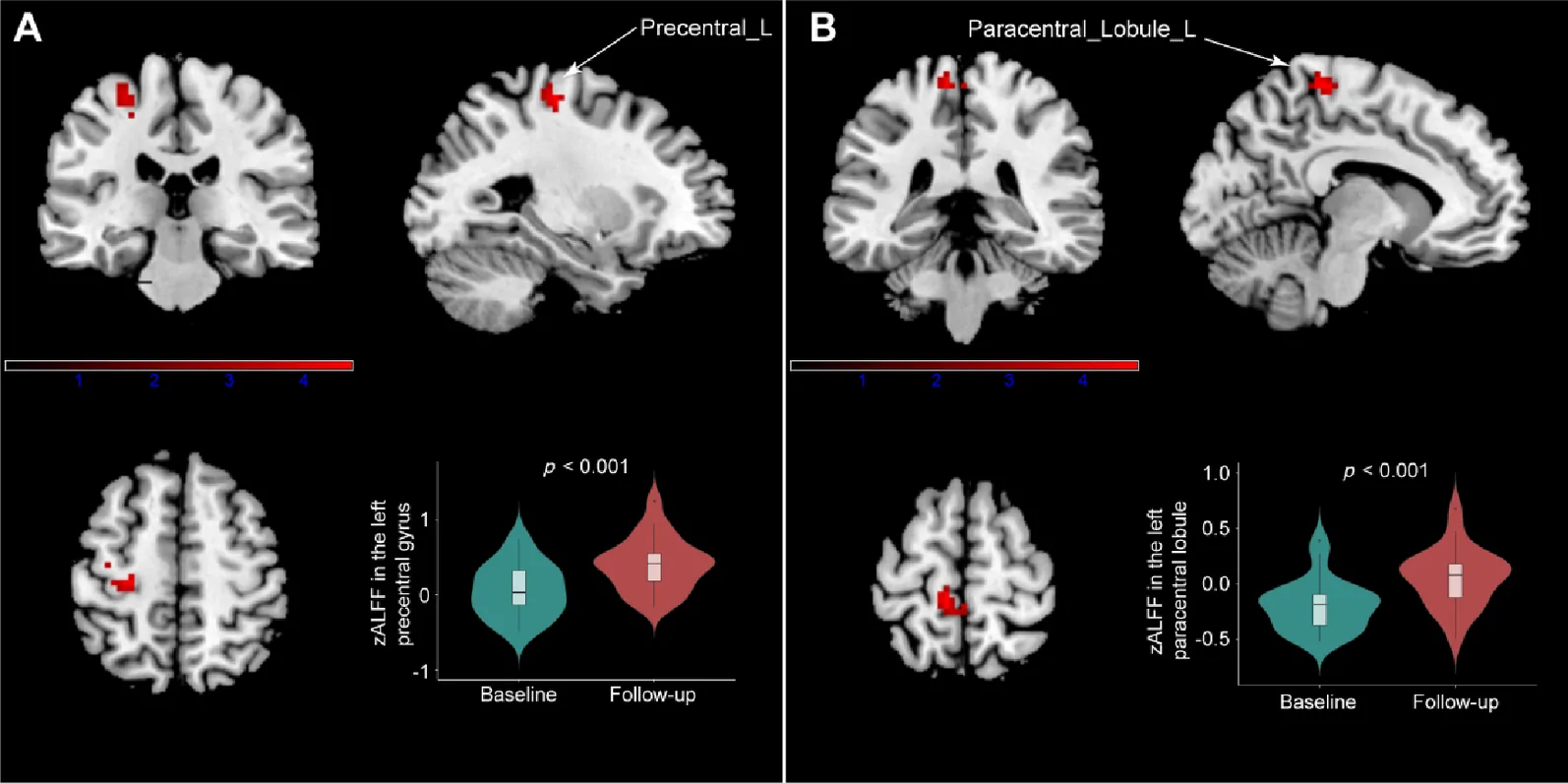
Longitudinal ALFF changes in SIS patients. The map highlights cortical regions central to our primary hypothesis (see Table 4 for cerebellar findings). **A** Left precentral gyrus showing increased ALFF at the 3-month follow-up compared with baseline (paired t-test, GRF-corrected, voxel-level *p* < 0.005, cluster-level *p* < 0.05), and violin plot illustrating longitudinal changes in mean ALFF values within the same region (paired t-test). **B** Left paracentral lobule showing increased ALFF at the 3-month follow-up compared with baseline (paired t-test, GRF-corrected, voxel-level *p* < 0.005, cluster-level *p* < 0.05), and violin plot illustrating longitudinal changes in mean ALFF values within the same region (paired t-test)

Consistent with our hypothesis, the left precentral gyrus, which showed reduced ALFF at baseline as part of a larger cluster extending from the postcentral gyrus, exhibited a significant increase in ALFF at the 3-month follow-up (peak MNI coordinate: -27, -24, 60). Additionally, increased ALFF was observed in the left paracentral lobule (peak MNI coordinate: -9, -33, 66). While multiple cerebellar regions exhibited elevated ALFF at baseline, the right cerebellar Crus II demonstrated a significant decrease in ALFF at the 3-month follow-up (GRF-corrected, voxel-level *p* < 0.005, cluster-level *p* < 0.05; Table 4; Fig. 3A, B).

**Table 4:** Changes in regional ALFF from baseline to the 3-month follow-up

Brain regions (AAL)	Number of voxels	MNI coordinate (x, y, z)	Peak T value
Precentral_L	31	(-27, -24, 60)	4.6272
Paracentral_Lobule_L	36	(-9, -33, 66)	4.7332
Cerebelum_Crus2_R	48	(48, -51, -42)	-5.9514

AAL, automated anatomical labeling; MNI, Montreal Neurological Institute; Precentral_L, left precentral gyrus; Paracentral_Lobule_L, left paracentral lobule; Cerebelum_Crus2_R, right cerebellar crus II

### Longitudinal changes in FC

**Fig. 4:**
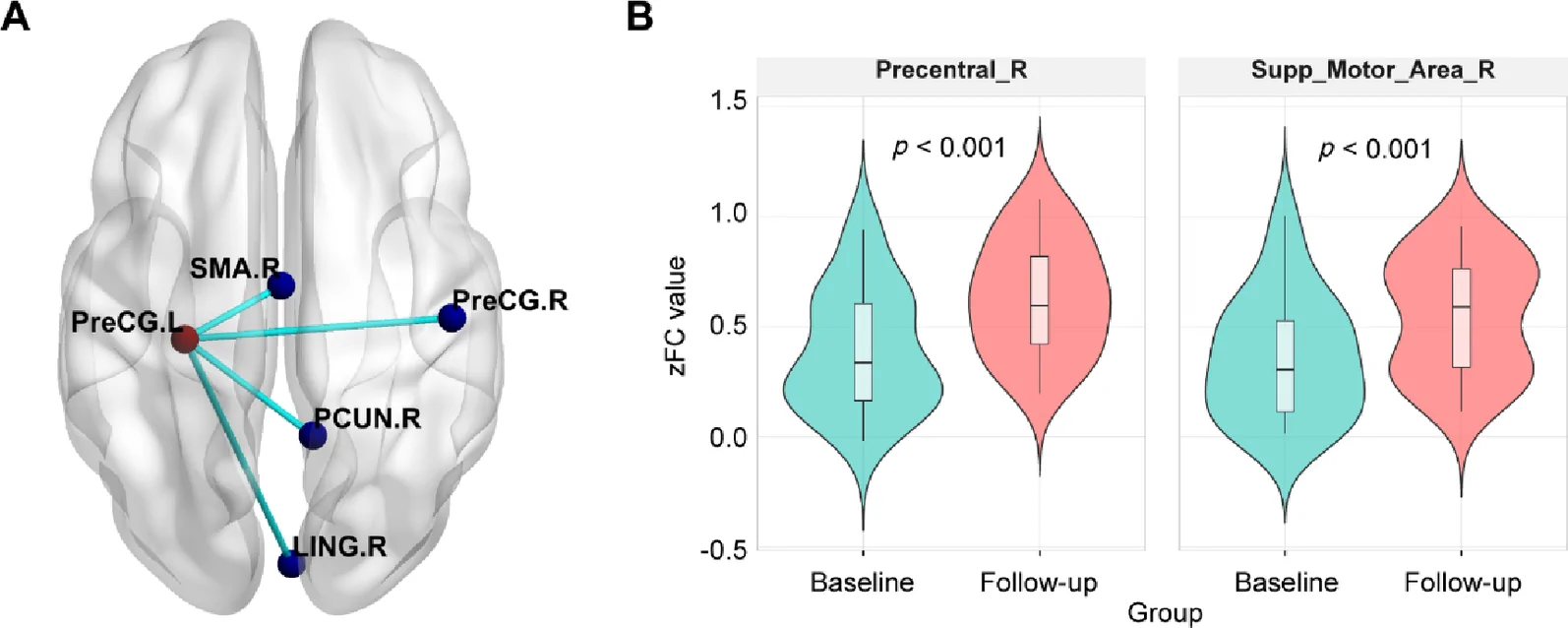
Longitudinal changes in FC seeded by regions with altered ALFF. This figure focuses on cortical regions (see Table 5 for cerebellar findings). **A** The seed region (left precentral gyrus) is shown as a red sphere. Blue spheres indicate brain regions showing significantly increased FC with the seed at the 3-month follow-up compared with baseline, including the right precentral gyrus, right supplementary motor area, right precuneus, and right lingual gyrus. Statistical analysis was performed using a paired t-test with GRF correction (voxel-level *p* < 0.005, cluster-level *p* < 0.05). **B** Within-group longitudinal comparison of mean FC values between the seed region and two representative motor-related regions—the right precentral gyrus and right supplementary motor area (paired t-test). FC changes in other regions (right precuneus and right lingual gyrus) are not displayed in this figure

To further investigate longitudinal changes in FC of brain regions showing significant ALFF differences, whole-brain FC analyses were performed using the left precentral gyrus (peak MNI coordinates: -27, -24, 60) and the left paracentral lobule (peak MNI coordinates: -9, -33, 66) as seed regions. Paired t-test revealed that, at the 3-month follow-up, the left precentral gyrus exhibited significantly increased FC with its homologous region (i.e., the right precentral gyrus). Additionally, this seed region showed significantly increased FC with the right supplementary motor area, right precuneus, right lingual gyrus, and right cerebellar lobules IV-V (GRF-corrected, voxel-level *p* < 0.005, cluster-level *p* < 0.05; Table 5; Fig. 4A, B). No significant changes in FC were observed in the whole-brain analysis using the left paracentral lobule as the seed region.

**Table 5:** Significant FC changes from baseline to 3-month follow-up

Brain regions (AAL)	Number of voxels	MNI coordinate (x, y, z)	Peak T value
Precentral_R	149	(48, -18, 60)	5.6399
Supp_Motor_Area_R	62	(0, -9, 48)	4.6297
Precuneus_R	72	(9, -51, 57)	4.7743
Lingual_R	72	(3, -87, -9)	5.0022
Cerebelum_4_5_R	219	(21, -51, -15)	4.9466

Seed region: left precentral gyrus. AAL, automated anatomical labeling; MNI, Montreal Neurological Institute; Precentral_R, right precentral gyrus; Supp_Motor_Area_R, right supplementary motor area; Precuneus_R, right precuneus; Lingual_R, right lingual gyrus; Cerebelum_4_5_R, right cerebellar lobules IV-V

### Correlation of longitudinal ALFF/FC changes in significant brain regions with clinical improvements

**Fig. 5:**
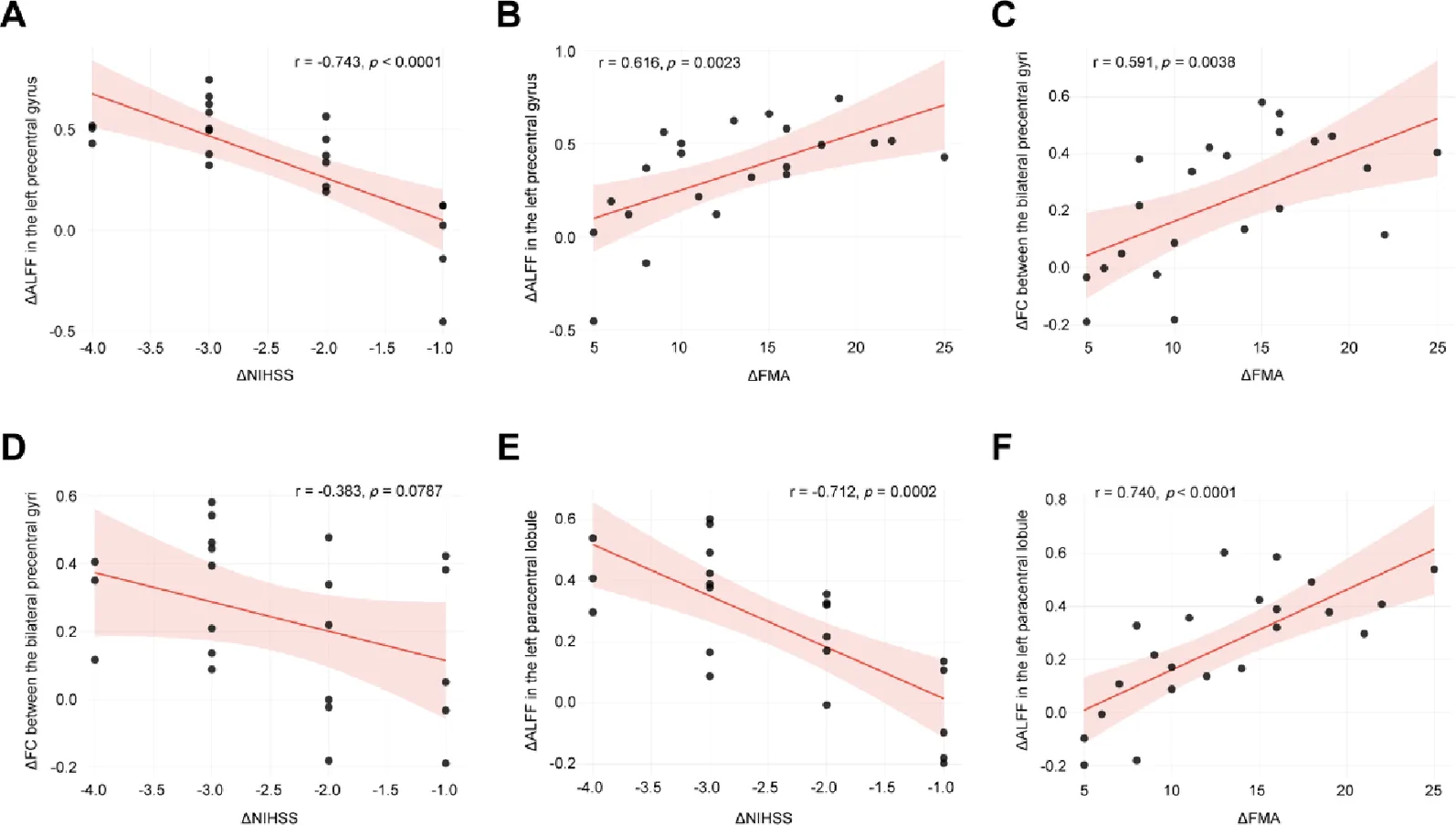
Correlations between longitudinal changes in ALFF/FC and clinical scores.** A** ΔNIHSS vs. ALFF change in the left precentral gyrus (*r* = -0.743, *p* < 0.0001). **B** ΔFMA vs. ALFF change in the left precentral gyrus (*r* = 0.616, *p* = 0.0023). **C** ΔFMA vs. FC change between the bilateral precentral gyri (*r* = 0.591, *p* = 0.0038). **D** ΔNIHSS vs. FC change between the bilateral precentral gyri (*r* = -0.383, *p* = 0.0787). **E** ΔNIHSS vs. ALFF change in the left paracentral lobule (*r* = -0.712, *p* = 0.0002). **F** ΔFMA vs. ALFF change in the left paracentral lobule (*r* = 0.740, *p* < 0.0001). All correlations shown, except that in **D**, survived Bonferroni correction (corrected threshold *p* < 0.0042)

Longitudinal changes in ALFF in the left precentral gyrus correlated with changes in both clinical scores: negatively with ΔNIHSS (*r* = -0.743, *p* < 0.0001; Fig. 5A) and positively with ΔFMA (*r* = 0.616, *p* = 0.0023; Fig. 5B). Similarly, changes in interhemispheric FC between the bilateral precentral gyri correlated positively with ΔFMA (*r* = 0.591, *p* = 0.0038; Fig. 5C) but not with ΔNIHSS (*r* = -0.383, *p* = 0.0787; Fig. 5D). Furthermore, ALFF changes in the left paracentral lobule correlated with changes in clinical scores: negatively with ΔNIHSS (*r* = -0.712, *p* = 0.0002; Fig. 5E) and positively with ΔFMA (*r* = 0.740, *p* < 0.0001; Fig. 5F). All correlations that were significant at the uncorrected threshold (*p* < 0.05) survived Bonferroni correction (corrected threshold *p* < 0.0042).

FC changes between the left precentral gyrus and the right supplementary motor area showed a negative correlation with ΔNIHSS (*r* = -0.528, *p* = 0.0116; Supplementary Fig. 1A) and a positive correlation with ΔFMA (*r* = 0.574, *p* = 0.0052; Supplementary Fig. 1B), but these did not survive Bonferroni correction. No significant correlations with either ΔNIHSS or ΔFMA (uncorrected *p* < 0.05) were observed for FC changes between the left precentral gyrus and the right precuneus or right lingual gyrus (Supplementary Fig. 1C–F).

### Gene expression patterns associated with longitudinal changes in ALFF

**Fig. 6:**
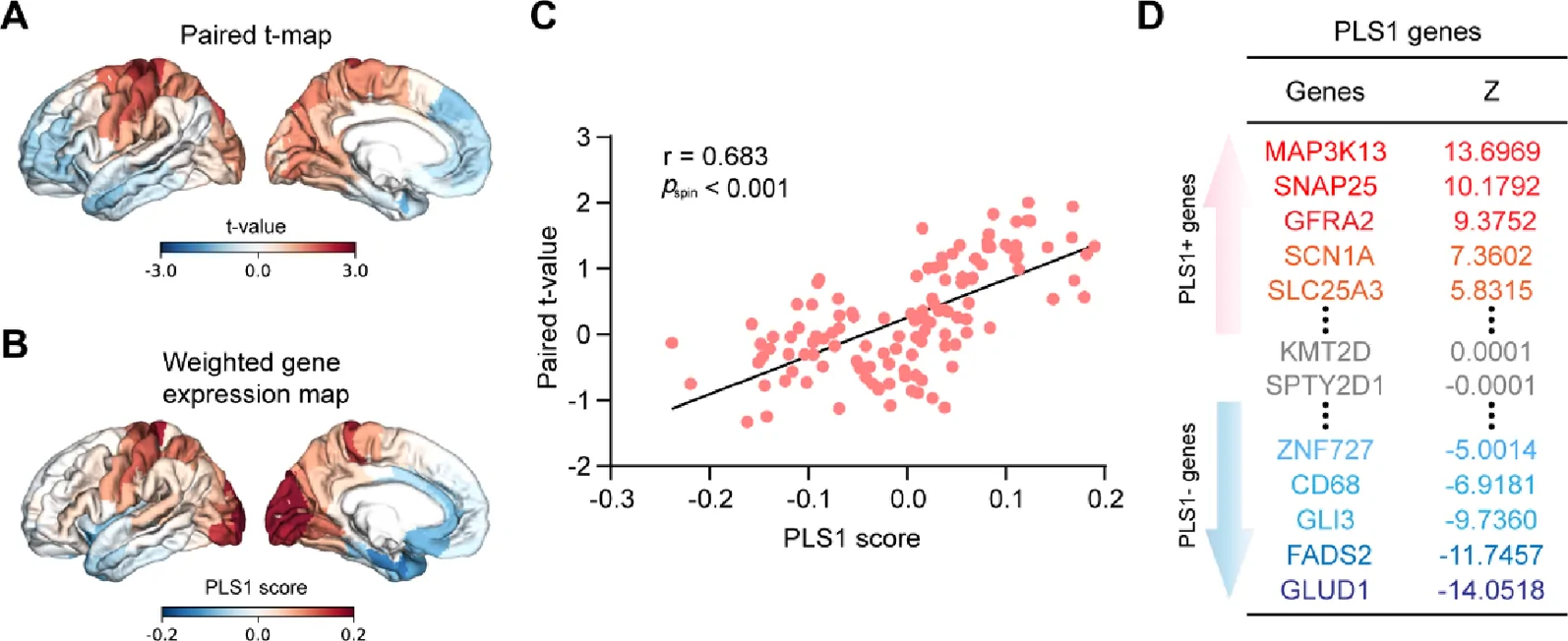
Transcriptomic signature associated with longitudinal regional ALFF changes. **A** Paired t-map illustrating longitudinal regional ALFF changes in the left hemisphere. **B** Weighted gene expression map showing regional PLS1 scores in the left hemisphere. **C** Spatial correlation between regional PLS1 scores and ALFF changes (paired t-map) in the left hemisphere of SIS patients; scatter plot shows a significant positive correlation (*r* = 0.683, *p*_spin_ < 0.001). **D** Ranking genes by Z-scores identified 1,613 PLS1+ genes (Z > 5) and 1,619 PLS1− genes (Z < −5), all with *p*_FDR_ < 0.0001

PLS regression was performed to identify gene expression patterns spatially correlated with longitudinal ALFF changes in the left hemisphere of SIS patients (Fig. 6A, B). PLS1, which constituted the linear combination of gene expression most strongly associated with regional ALFF changes, accounted for 42.99% of the total variance (permutation test, *p*_spin_ < 0.0001) and was therefore retained for subsequent analysis. Notably, the PLS1-weighted gene expression map showed a significant positive spatial correlation with the ALFF paired t-map (*r* = 0.683, *p*_spin_ < 0.001; Fig. 6C), indicating that PLS1+ genes were upregulated in brain regions where ALFF increased at the 3-month follow-up compared with baseline, whereas PLS1− genes were upregulated in regions where ALFF decreased. Based on the univariate one-sample Z-test, genes with significant PLS1 weights were identified and divided into two sets: 1,613 PLS1+ genes (Z > 5) and 1,619 PLS1− genes (Z < −5), each achieving an FDR-corrected *p* < 0.0001 (Supplementary Table 2; Fig. 6D). Collectively, these genes delineate a transcriptomic signature associated with longitudinal ALFF changes (3-month follow-up vs. baseline) in SIS patients.

### Functional enrichment of PLS1 genes

**Fig. 7:**
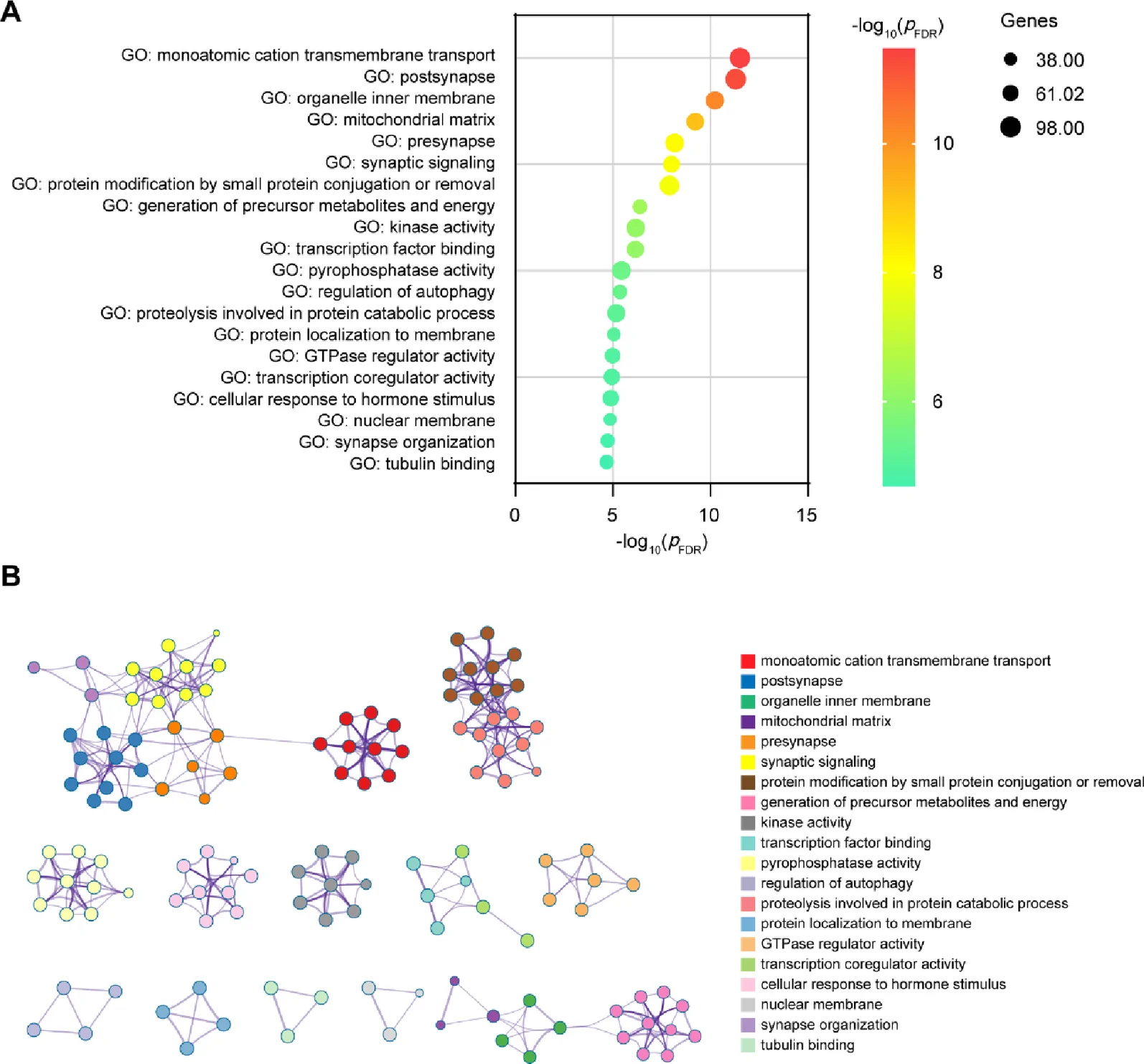
GO and KEGG enrichment analysis of PLS1+ genes.** A** Bubble plot displaying the top 20 significantly enriched GO terms for PLS1+ genes. No KEGG pathways were significantly enriched. Bubble color represents -log₁₀(FDR-corrected *p*-value), and bubble size indicates the number of genes assigned to each term. **B** Network visualization of enriched GO terms via Metascape. GO, Gene Ontology; KEGG, Kyoto Encyclopedia of Genes and Genomes

We performed GO and KEGG pathway enrichment analyses separately for the PLS1+ and PLS1− gene sets. Following FDR correction, the PLS1+ genes showed significant enrichment for a series of terms closely related to synaptic structure and signaling, metabolic processes, and cellular regulation. Specifically, for Cellular Component, they were enriched in items such as “presynapse”, “postsynapse”, “organelle inner membrane”, and “mitochondrial matrix”. For Biological Process, enrichment was observed in processes including “synaptic signaling”, “synapse organization”, “monoatomic cation transmembrane transport”, “generation of precursor metabolites and energy”, “protein localization to membrane”, and “regulation of autophagy”. For Molecular Function, the enriched terms primarily involved “kinase activity”, “GTPase regulator activity”, and “tubulin binding”. However, no pathways showed significant enrichment in the KEGG analysis (Fig. 7A, B).

In contrast, the PLS1− genes were mainly enriched in GO terms covering broader functional categories, including cell adhesion and membrane structure organization, metabolism and stress response, negative regulation of immune responses, negative regulation of cell proliferation and differentiation, gliogenesis, and development of multiple tissues and organs (e.g., sensory organs, brain, heart). Furthermore, KEGG pathway analysis revealed their significant enrichment in the Coronavirus disease (COVID-19) pathway (Supplementary Fig. 2A, B).

### Cell type signature of PLS1+ genes

**Fig. 8:**
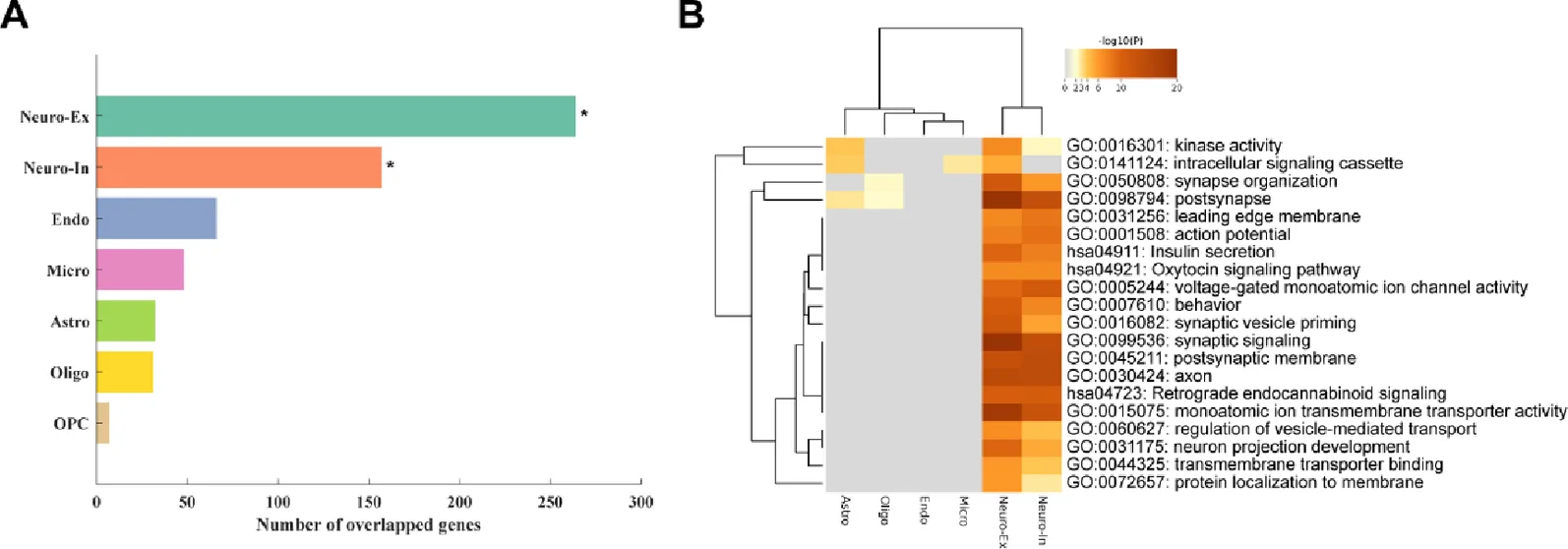
Cell type signature of PLS1+ genes. **A** Bar plot showing the number of overlapping genes between the PLS1+ genes and each cell type-specific gene set. Significant overlaps, indicated by asterisks, were observed for excitatory neurons (264 genes, FDR-corrected *p*_perm_ < 0.001) and inhibitory neurons (157 genes, FDR-corrected *p*_perm_ < 0.001). **B** Heatmap of enriched GO terms and KEGG pathways for PLS1+ genes across different cell types. Color intensity represents -log₁₀(*p*-value), with darker colors indicating higher statistical significance. Neuro_Ex: Excitatory neurons; Neuro_In: Inhibitory neurons; Endo: Endothelial cells; Micro: Microglia; Astro: Astrocytes; Oligo: Oligodendrocytes; OPC: Oligodendrocyte precursor cells

To further investigate the cell-type specificity of genes associated with longitudinal ALFF changes, we assigned the PLS1+ genes to seven canonical cell types. The PLS1+ genes showed significant overlap with excitatory (264 genes, *p*_perm_ < 0.001 after FDR correction) and inhibitory neurons (157 genes, *p*_perm_ < 0.001 after FDR correction) (Fig. 8A). Cell type-specific enrichment analyses showed that, in both neuronal types, PLS1+ genes were enriched in GO terms related to synaptic structure and function, including “synapse organization”, “postsynapse”, “postsynaptic membrane”, “axon”, “synaptic signaling”, “synaptic vesicle priming”, “action potential”, “voltage-gated monoatomic ion channel activity”, “monoatomic ion transmembrane transporter activity”, and “regulation of vesicle-mediated transport”. KEGG analysis further revealed enrichment in “Retrograde endocannabinoid signaling”, “Oxytocin signaling pathway”, and “Insulin secretion” (Fig. 8B).

## Discussion

Understanding the neural mechanisms underlying post-stroke motor recovery in the context of routine clinical management is critical for advancing rehabilitation. To our knowledge, this study is the first to delineate the dynamic, multiscale landscape of recovery after SIS by integrating longitudinal rs-fMRI with AHBA transcriptomic data. We found that recovery was characterized by a significant increase in spontaneous neural activity (ALFF) in the contralesional (i.e., left) precentral gyrus, accompanied by enhanced interhemispheric FC with its ipsilesional homologue. Longitudinal ALFF changes in this node positively correlated with motor improvement. To further explore the potential molecular basis of plasticity in this node, we correlated longitudinal ALFF changes across the left hemisphere with AHBA gene expression data and identified PLS1+ genes that were positively associated with these ALFF changes. These genes were significantly involved in excitatory and inhibitory neurons and were enriched in neuronal processes related to synaptic function. Given that the contralesional precentral gyrus showed the most significant ALFF increases, these findings provide multiscale evidence, spanning from macroscopic neuroimaging to the molecular level, for understanding the neuroplasticity of the contralesional precentral gyrus in motor recovery after SIS.

### Functional reorganization of the contralesional precentral gyrus: from acute inhibition to compensatory activation

The “interhemispheric competition model” posits that stroke disrupts the normal inhibitory balance between the two hemispheres, leading to relative hyperactivity in the contralesional hemisphere [12]. However, the present study found that after acute SIS, neural activity in the contralesional precentral gyrus unexpectedly decreased. This early inhibitory phenomenon is difficult to explain by the above classic model but instead aligns with the concept of “diaschisis”. The latter states that a focal lesion can lead to reduced excitability or “functional stillstand” in remote, anatomically intact brain regions. Notably, cortical diaschisis induced by a subcortical lesion (such as SIS in our study) has a more explicit correlation with patients’ behavioral deficits [45]. This provides a direct pathophysiological basis for understanding the early contralesional inhibition observed in our study.

A key feature of “diaschisis” is its dynamic reversibility over time [45], which provides a potential time window for subsequent functional reorganization. Consistent with this, the present study found that activity in the contralesional precentral gyrus significantly recovered from its acute inhibitory state by the 3-month follow-up. This not only confirms the reversible process of diaschisis but also suggests that the recovery of activity indicates this brain region may be released from its initial inhibition, further recruited, and transformed into a positive, compensatory role [7, 10, 12, 46]. Indeed, increased activity in the contralesional motor cortex has been associated with motor recovery [11], and brain-computer interface training can specifically enhance activity in this region, accompanied by functional improvement [21]. One interpretation is that when the ipsilesional motor pathway is damaged, the contralesional precentral gyrus may participate in motor control of the paretic limb by strengthening its internal excitability and increasing the activity of ipsilateral corticospinal projections, particularly in patients with moderate-to-severe stroke [14, 47]. In our SIS patients with marked motor impairment, we found that motor recovery (FMA improvement) at the 3-month follow-up was positively correlated with increased ALFF in the contralesional precentral gyrus. This finding is consistent with the above interpretation, suggesting that the enhanced excitability observed in this region during the subacute phase may reflect a compensatory process following SIS.

### Reorganization of interhemispheric FC in the precentral gyri

Our FC findings suggest a compensatory role of the contralesional hemisphere after SIS. We found that enhanced FC between the bilateral precentral gyri at 3 months post-stroke correlated with improvement in FMA scores. This pattern is consistent with the “bilateral motor network reorganization” framework [48]. According to this view, post-stroke motor recovery may involve a dynamic reorganization of interhemispheric connectivity: an initial decrease in FC between homologous regions is associated with behavioral deficits, followed by a gradual reestablishment and strengthening of these connections, a process that may provide a neural basis for functional recovery [48–50].

Building on this, our results challenge the traditional “interhemispheric competition” hypothesis regarding the contralesional hemisphere’s role in the subacute phase. This hypothesis posits that the intact hemisphere may excessively inhibit the injured hemisphere, thereby impeding recovery [12, 51]. In contrast, our data suggest that interhemispheric interactions during this stage may be more supportive than competitive. This observation aligns with recent findings by Fokas et al., who also observed no clear detrimental effects of inhibition from the intact hemisphere in mild-to-moderate subacute stroke. In some patients, stronger inhibition even correlated with better motor function of the affected upper limb, implying a supportive role [52]. Together, these findings suggest that the contralesional hemisphere’s role during the subacute phase may be more complex than traditionally assumed. Consequently, rehabilitation strategies aimed at reducing interhemispheric inhibition from the contralesional hemisphere may require more careful evaluation of their general applicability at this stage.

### Potential critical role of synaptic plasticity in the contralesional precentral gyrus for post-stroke motor recovery

A key innovation of this study lies in the integrative analysis linking longitudinal post-stroke neuroimaging changes to hemispheric gene expression profiles. We found that in brain regions exhibiting elevated ALFF at 3 months post-stroke, particularly in the contralesional precentral gyrus, the PLS1+ gene set was overexpressed. Functional enrichment analysis revealed that these genes were significantly associated with synaptic structure and function. Together, these findings point to synaptic plasticity as a potential critical mechanism underlying the macroscopic functional reorganization reflected by ALFF changes. Accumulating evidence indicates that motor recovery after stroke is a dynamic process driven by neural plasticity, involving multi-scale adaptive reorganization from synapses to whole-brain networks [12, 53–55]. Notably, animal model studies have demonstrated that post-stroke neural plasticity extends far beyond the peri-infarct region. Specifically, the corticospinal projection from the contralesional motor cortex to the denervated cervical spinal cord exhibits structural plasticity (axonal sprouting), and such plastic changes are positively correlated with the level of motor recovery [56–58]. Inverse validation experiments further support this causal relationship, showing that selective and temporary blockade of these newly sprouted fibers suppresses recovered limb function [59]. Furthermore, while improving fine motor walking function in rats, constraint-induced movement therapy primarily induces neuroplastic changes in the contralesional motor cortex. These changes encompass the recruitment of additional neurons to form new networks controlling the paretic forelimb, as well as a specific increase in synaptic density in this region [60]. Integrating evidence from these animal studies, our gene expression–neuroimaging association analysis further strengthens the role of synaptic plasticity in the contralesional precentral gyrus as a key correlate of post-stroke motor recovery in humans.

### Synaptic plasticity-mediated excitation–inhibition balance remodeling: an integrative framework for post-stroke motor recovery

The PLS1+ gene set associated with longitudinal ALFF changes was not ubiquitously expressed across all brain cell types but was specifically enriched in excitatory and inhibitory neurons, and showed significant enrichment for the retrograde endocannabinoid signaling pathway. This suggests that post-stroke functional recovery may depend on the re-establishment of excitation–inhibition (E–I) balance [61, 62], with endocannabinoid signaling potentially serving as a key mechanism underlying this process. Consistently, the observed normalization of resting-state ALFF may macroscopically reflect the reorganization and re-optimization of neural circuits based on such molecular mechanisms. Retrograde endocannabinoid signaling is a core system for dynamically regulating E–I balance [63, 64]. When postsynaptic neuronal activity is enhanced, released endocannabinoids act retrogradely on presynaptic cannabinoid receptor 1 (CB1) to selectively inhibit the release of both glutamate and GABA. This activity-dependent negative feedback mitigates excitotoxicity and, when necessary, produces transient disinhibition, thereby creating precise local conditions for synaptic plasticity events such as long-term potentiation or dendritic spine formation [64–66]. Here, excitatory synaptic plasticity underlies motor learning [67], while inhibitory neurons play a critical role in fine-tuning network excitability and synchrony to ensure the stability and controllability of newly formed circuits [68]. Their synergy establishes a novel, adaptive network equilibrium. This mechanism aligns with the requirement for precise E–I balance in both motor learning [69] and post-stroke cortical reorganization [61, 62]. In summary, this study provides transcriptome-level evidence suggesting that re-establishment of E–I balance via endocannabinoid signaling may constitute a key synaptic mechanism for post-stroke motor recovery.

### Limitations

This study has several limitations. First, the limited sample size constrained the statistical power and hindered reliable subgroup analyses based on clinical features. Therefore, the current findings should be regarded as preliminary evidence, and their robustness needs to be verified in larger, independent cohorts. Second, restricted by the availability of AHBA data, the transcriptomic-neuroimaging correlation analysis was conducted only in the left hemisphere. As all enrolled patients had right-hemisphere subcortical stroke, the present work characterizes the molecular correlates of the contralesional hemisphere but did not assess the ipsilesional hemisphere. Moreover, the correlative nature of the analysis precludes direct causal inference. Third, the core analytical strategy of spatially correlating transcriptomic data with neuroimaging data entails inherent data heterogeneity. Gene expression data were derived from a limited number of postmortem brain tissue samples from donors without known neurological disorders, whereas the neuroimaging data came from an independent cohort of living stroke patients. Although certain gene expression patterns are conserved across individuals—providing a rationale for cross‑subject correlation—differences in donor age, sex, and genetic background remain potential confounders that warrant caution in interpretation. Fourth, all participants received routine clinical care, but rehabilitation dosage varied across individuals and was not quantified. Consequently, the observed neural changes likely reflect the combined effects of spontaneous and intervention‑induced neuroplasticity [70], which limits the precise delineation of their independent contributions. Future studies should employ more rigorous designs (e.g., comparing different doses of standardized rehabilitation) to explore the relative contributions of these two forms of plasticity. Finally, the follow-up window was limited to 3 months post-stroke. Because the full manifestation or evolution of certain motor deficits may be delayed well beyond this timeframe [71], the present study primarily provides insights into the mechanisms operating in the subacute phase (i.e., the first 3 months) after stroke. Extending the follow-up period (e.g., to at least 6 months) and incorporating finer-grained behavioral assessments will be needed to investigate the longer-term evolution of these neural mechanisms.

## Conclusion

This study integrated longitudinal neuroimaging and AHBA transcriptomic data to preliminarily explore the potential mechanisms underlying motor recovery after SIS. From baseline to the 3‑month follow‑up, motor recovery was found to be closely associated with increased spontaneous neural activity in the contralesional precentral gyrus and strengthened FC with its ipsilesional homologous region. Further transcriptomic‑neuroimaging correlation analysis demonstrated that these longitudinal changes in brain activity were spatially correlated with a specific gene expression profile enriched in excitatory/inhibitory neurons and closely related to synaptic plasticity. By linking macroscopic and microscopic features, this study provides multiscale evidence for the key role of the contralesional precentral gyrus during the subacute phase. More importantly, the synergistic pattern of functional enhancement in this region and strengthened interhemispheric FC highlights the potentially “supportive” role of interhemispheric interactions at this stage, suggesting that future therapeutic strategies may need to consider stage‑specific refinements regarding interventions targeting interhemispheric inhibition.

## Supplementary Information

Below is the link to the electronic supplementary material.

Supplementary Material 1.

Supplementary Material 2.

## Data Availability

Data supporting the findings of this study are available from the corresponding author upon reasonable request.

## Abbreviations

rs-fMRI: Resting-state functional magnetic resonance imaging
ALFF: Amplitude of low-frequency fluctuations
FC: Functional connectivity
AHBA: Allen Human Brain Atlas
NIHSS: National Institutes of Health Stroke Scale
FMA: Fugl-Meyer Assessment
SIS: Subcortical ischemic stroke
PLS: Partial least squares
FDR: False discovery rate
GRF: Gaussian random field
FD: Framewise displacement
MNI: Montreal Neurological Institute
GO: Gene Ontology
KEGG: Kyoto Encyclopedia of Genes and Genomes
