# Supplementary material for: Functional reorganization of the contralesional precentral gyrus following acute subcortical ischemic stroke and its association with gene expression profile
Source: J Neuroeng Rehabil. 2026 Jun 12;23:241. doi: 10.1186/s12984-026-02048-w (PMC13487995; doi:10.1186/s12984-026-02048-w)
Supplement: Supplementary file 1 [file 12984_2026_2048_MOESM1_ESM.docx]

**Supplementary Table 1. Individual clinical characteristics of 22 patients with right-hemispheric subcortical ischemic stroke**

| Patient | Paresis pattern | Lesion location | Baseline FMA (total) | Baseline  NIHSS-motor arm | Baseline  NIHSS-motor leg | ΔFMA  (total) | | ΔNIHSS  (total) |
| --- | --- | --- | --- | --- | --- | --- | --- | --- |
| 01 | Hemiparesis | IC | 77 | 2 | 1 | 5 | | -1 |
| 02 | Hemiparesis | IC, CR | 73 | 2 | 1 | | 21 | -4 |
| 03 | Hemiparesis | BG, CR | 82 | 1 | 1 | | 16 | -3 |
| 04 | Hemiparesis | IC | 91 | 1 | 1 | | 9 | -2 |
| 05 | Hemiparesis | BG, IC | 66 | 2 | 2 | | 14 | -3 |
| 06 | Hemiparesis | BG, IC | 81 | 1 | 1 | | 18 | -3 |
| 07 | Hemiparesis | IC, CR | 89 | 1 | 1 | | 10 | -3 |
| 08 | Hemiparesis | BG, CR | 76 | 2 | 1 | | 5 | -1 |
| 09 | Hemiparesis | BG, IC | 74 | 2 | 1 | | 22 | -4 |
| 10 | Hemiparesis | BG, CR | 72 | 2 | 2 | | 12 | -1 |
| 11 | Hemiparesis | IC | 81 | 1 | 1 | | 19 | -3 |
| 12 | Hemiparesis | BG, IC, CR | 62 | 2 | 2 | | 16 | -2 |
| 13 | Hemiparesis | BG, CR | 91 | 1 | 1 | | 6 | -2 |
| 14 | Faciobrachial hemiparesis | IC | 92 | 1 | 0 | | 8 | -2 |
| 15 | Hemiparesis | BG, IC, CR | 72 | 2 | 1 | | 8 | -1 |
| 16 | Hemiparesis | IC | 88 | 1 | 1 | | 10 | -2 |
| 17 | Hemiparesis | BG | 85 | 1 | 1 | | 15 | -3 |
| 18 | Hemiparesis | BG, IC | 84 | 1 | 1 | | 13 | -3 |
| 19 | Hemiplegia | BG, CR | 36 | 3 | 3 | | 25 | -4 |
| 20 | Hemiparesis | BG, CR | 68 | 2 | 2 | | 7 | -1 |
| 21 | Hemiparesis | BG, CR | 82 | 1 | 1 | | 11 | -2 |
| 22 | Hemiparesis | BG, IC | 83 | 1 | 1 | | 16 | -3 |

Abbreviations: BG, basal ganglia; CR, corona radiata; IC, internal capsule; FMA, Fugl-Meyer Assessment (total score, range 0–100); NIHSS, National Institutes of Health Stroke Scale; NIHSS-motor arm, upper extremity score (0 = no drift for 10 s, 1 = drift, 2 = falls within 10 s with some antigravity effort, 3 = no antigravity effort, 4 = no movement); NIHSS-motor leg, lower extremity score (0 = no drift for 5 s, 1 = drift, 2 = falls within 5 s with some antigravity effort, 3 = no antigravity effort, 4 = no movement).

**Supplementary Fig. 1. Correlations between longitudinal changes in other FC metrics and clinical scores.** (**A**) ΔNIHSS vs. FC change between the left precentral gyrus and the right supplementary motor area (r = -0.528, *p* = 0.0116). (**B**) ΔFMA vs. FC change between the left precentral gyrus and the right supplementary motor area (r = 0.574, *p* = 0.0052). (**C, D**) FC change between the left precentral gyrus and the right precuneus vs. (C) ΔNIHSS and (D) ΔFMA: no significant correlation. (**E, F**) FC change between the left precentral gyrus and the right lingual gyrus vs. (E) ΔNIHSS and (F) ΔFMA: no significant correlation.

Note: All *p*-values are uncorrected. Correlations in (A) and (B) did not survive Bonferroni correction (corrected threshold *p* < 0.0042).

**Supplementary Fig. 2. GO and KEGG enrichment analysis of PLS1− genes.** (**A**) Bar plot of the top 20 significantly enriched GO terms and significantly enriched KEGG pathways for PLS1− genes. Bar height represents -log₁₀(*p*-value). (**B**) Network visualization of enriched GO terms and KEGG pathways generated using Metascape. GO, Gene Ontology; KEGG, Kyoto Encyclopedia of Genes and Genomes.
